# Obesity May Not Induce Dynamic Stability Disadvantage during Overground Walking among Young Adults

**DOI:** 10.1371/journal.pone.0169766

**Published:** 2017-01-13

**Authors:** Zhong-Qi Liu, Feng Yang

**Affiliations:** 1 School of Biological Science and Medical Engineering, Beihang University, Beijing, China; 2 Department of Kinesiology, University of Texas at El Paso, El Paso, TX, United States of America; Charite Universitatsmedizin Berlin, GERMANY

## Abstract

Obesity has been related to postural instability during static standing. It remains unknown how obesity influences stability during dynamic movements like gait. The primary aim of this study was to investigate the effects of obesity on dynamic gait stability control in young adults during gait. Forty-four young adults (21 normal-weight and 23 obese) participated in this study. Participants walked five times at their self-selected gait speeds on a linear walkway. Their full-body kinematics were gathered by a motion capture system. Compared with normal-weight group, individuals with obesity walked more slowly with a shorter but wider step. People with obesity also spent an elongated double stance phase than those with normal weight. A reduced gait speed decreases the body center of mass’s velocity relative to the base of support, leading to a reduction in dynamic stability. On the other hand, a shortened step in accompanying with a less backward-leaning trunk has the potential to bring the center of mass closer to the base of support, resulting in an increase in dynamic stability. As the result of these adaptive changes to the gait pattern, dynamic gait stability among people with obesity did not significantly differ from the one among people with normal weight. Obesity seems to not be inducing dynamic stability disadvantage in young adults during level overground walking. These findings could provide insight into the mechanisms of stability control among people affected by obesity during dynamic locomotion.

## Introduction

Obesity is a major public health issue and the incidence of obesity rises at a staggering rate [1]. Proper balance maintenance and stability control are essential for activities of human daily living. The excessive body mass from obesity may exert adverse structural and functional effects on human body, affecting body posture and balance control [2]. Given the inherent invert-pendulum nature of the human body, the extra mass may require more effort to stabilize the body. Additionally, the greater pressure values and larger contact areas beneath the feet among people with obesity may impair the sensory capability from the plantar mechanoreceptors which is of the essence for balance control [3]. Therefore, obesity may cause an elevated postural instability [4], increasing the risk of falls and injuries among obese [5]. To date, most studies reported the postural instability characterized by the center of pressure movement on both anteroposterior and mediolateral directions during quiet standing–a typical static task [6–8]. Nevertheless, the majority of real-life falls happen during locomotion [9] and the stability evaluated during static condition has shown poor ability to predict future falls [10]. It is, therefore, desired to investigate what effects obesity imposes on stability control during dynamic human locomotion.

**Fig 1:**
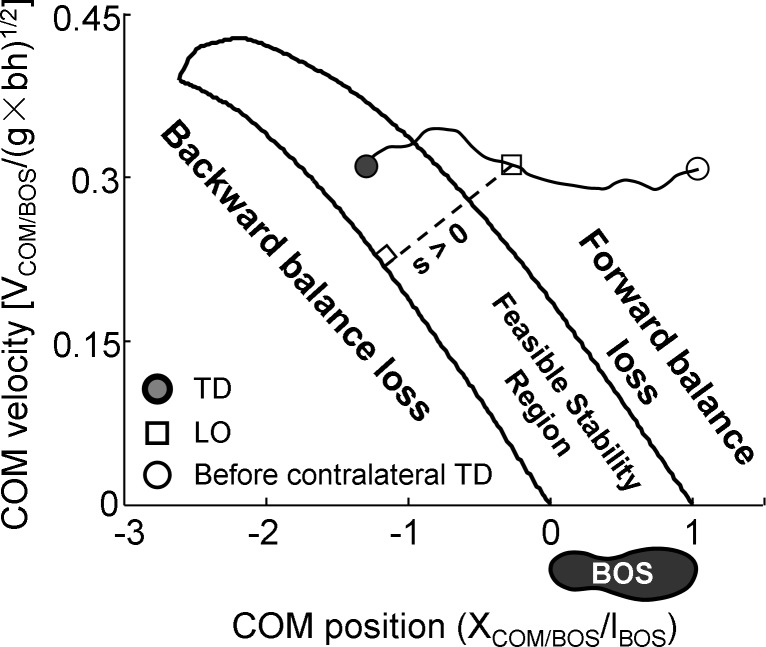
The feasible stability region. The illustration showing the feasible stability region (FSR) which is bounded by two borders: the limit against backward balance loss (the lower boundary) and the one against forward balance loss (the upper boundary). The stability measurement (*s*, the length of the thin solid line) indicates the magnitude of the instantaneous stability of the center of mass (COM) against backward balance loss, and is calculated as the shortest distance from the instantaneous COM motion state (i.e., the *x*- and *y*-coordinates represents the COM anteroposterior position and velocity, respectively) to the limit against backward balance loss. Also shown is a representative COM motion state trajectory of an overground walking (the thin line) progressing from the touchdown (TD, filled circle), through the contralateral foot liftoff (LO, square), and immediately prior to the contralateral foot TD (open circle). Position and velocity of the COM relative to the base of support (BOS) are *dimensionless* as a fraction of *l*_*BOS*_ and $\sqrt{g\times bh}$, respectively, where *l*_*BOS*_ represents the foot length, *g* is gravitational acceleration, and *bh* the body height. When the COM motion state is outside the FSR, the person is either backward instable (below the limit against backward balance loss) or forward instable (above the limit against forward balance loss). A recovery step becomes necessary to keep the person from falling either backward or forward.

Gait variability has been applied to quantify its stability. Based on the linear (such as the standard deviations of spatiotemporal gait parameters [11]) or nonlinear dynamics (such as the maximum Floquet multipliers [12] and Lyapunov exponents [13]) theory for cyclical movement, variability in kinematics is indicative of stability. Alternatively, based on the Feasible Stability Region theory (FSR), dynamic gait stability has been proposed to quantify one’s resistance to balance loss during gait [14, 15]. The FSR is comprised of two limits: the limit against backward balance loss and the one against forward balance loss (Fig 1). These two limits encompass all possible motion states (i.e., the combination of position and velocity) of the body center of mass (COM) relative to the base of support (BOS) which ensure a person to keep the balanced upright body posture during gait. If the COM motion state is within the FSR, one is in a stable state and would preserve body balance without changing the existing BOS. When the COM motion state is below the FSR (or the limit against backward balance loss), the person is in an unstable state because the COM has no sufficient forward momentum to carry it over the BOS when its velocity diminishes. The person must take a backward recovery step to keep the body from falling backward, encountering a backward balance loss. Conversely, a person whose COM motion state is above the FSR (or the limit against forward balance loss), s/he is also in an unstable state since the COM possesses excessive forward momentum that would move the COM anteriorly beyond the BOS when its velocity becomes zero, resulting a forward balance loss. Dynamic gait stability has been identified as a more accurate predictor of falls in comparison with those indices based on gait variability [16, 17]. Therefore, the examination of obesity’s effects on dynamic gait stability may uncover the mechanisms of obesity increasing fall risk.

Previous studies regarding the influence of obesity on gait parameters indicated that people with obesity have a slower gait speed with a shorter but wider step and a longer double stance compared to their normal-weight counterparts [18–20]. Based on the FSR theory, a shortened step brings the COM closer to the BOS [21] and in turn improves the dynamic stability against backward balance loss while a reduced gait speed has the potential to deteriorate the dynamic stability [14]. The opposing effects from these two factors on the dynamic stability could nullify the influence of each likely resulting in comparable stability between normal-weight and obese individuals. Nevertheless, the exact impact of obesity on dynamic stability remains to be determined.

The primary purpose of this study was to inspect how obesity affects dynamic gait stability among young adults. Given the opposite effects of slow gait speed and short step length on dynamic gait stability, we hypothesized that people with obesity would exhibit a comparable dynamic stability during gait with their normal-weight counterparts. The findings from this study could provide insights into the influences of obesity on dynamic stability control during human gait.

## Methods and Materials

### Participants

Young individuals with (*n* = 23) and without (*n* = 21) obesity participated in this study (Table 1). All participants were free of any clinically significant history of musculoskeletal disorders, neurological disorders, orthopedic conditions, and cardiovascular conditions. They gave their written consent for participation in the study approved by the Institutional Review Board at the University of Texas at El Paso.

**Table 1:** Demographic information in mean ± standard deviation for both normal-weight (or Normal, *n* = 21) and obese (or Obese, *n* = 23) groups.

Groups	Age (years)	Gender (female)	Height (m)	Mass (kg)	BMI (kg/m^2^)	BIA (fat %)	Leg length (m)	Standing width (m)
**Normal**	23.5 ± 4.0	14	1.64 ± 0.09	58.5 ± 11.0	21.7 ± 2.4	20.0 ± 5.9	0.87 ± 0.06	0.22 ± 0.04
**Obese**	24.9 ± 5.7	8	1.71 ± 0.10	102.9 ± 17.6	35.1 ± 3.9	37.3 ± 6.0	0.90 ± 0.05	0.24 ± 0.04
***p* value**	0.338	0.069^a^	0.018	< 0.001	< 0.001	< 0.001	0.107	0.060

BMI: body mass index.

The standing width was calculated as the mediolateral distance between two heels during a static standing calibration trail.

^a^: Fisher’s exact test was used.

Only those who were either normal-weight or obese were enrolled into the study. A person with the body mass index (BMI) between 18 and 25 kg/m^2^ was considered a normal-weight participant [5]. Whether a participant is obese was determined using two criteria. Specifically, for a male participant, his BMI must be no less than 30 kg/m^2^ and the body fat percentage should be equal to or greater than 25%. For a female participant, her BMI and body fat percentage must be at least 30 kg/m^2^ and 35%, respectively [22]. The bioelectrical impedance analysis was used to gauge the body fat percentage through a body composition analyzer (Tanita Corp., Japan). Such an inclusion criterion excluded overweight individuals who are between the clear obese and normal-weight participants in order to avoid any potential effects from overweight individuals on our findings.

### Experimental Protocol and Data Collection

After being measured for the basic demographic information, each participant was brought to a 14-m walkway, over which they walked five times at their self-selected speed. Full body kinematics data from 26 retro-reflective markers placed on the subjects’ body were gathered using an 8-camera motion capture system (Vicon, UK) at 120 Hz. The fifth trial was selected as the representative trial for analysis.

Marker position data were low-pass filtered at marker-specific cut-off frequencies (ranging from 4.5 to 9 Hz) using fourth-order, zero-lag Butterworth filters [23]. Locations of joint centers, heels, and toes were computed from the filtered marker positions. The timing of two characteristic and transient events in each gait cycle: touchdown (TD) and liftoff (LO), was identified from the foot kinematics. Temporal measures included the double (from TD to subsequent LO of the contralateral limb) and single (from LO to the following TD at the ipsilateral foot) stance phase times and the step time (from TD to the following TD of the contralateral side). Both the double and single stance phases were also represented as a percentage of the gait cycle. The cadence was determined as the reciprocal of the step time and expressed over one minute.

Spatial measurements consisted of the step length, step width, and trunk angle. Step length was calculated as the anteroposterior distance between the two heels at their TDs. The step width was the mediolateral distance between the heels at their TDs. It was suggested that spatial parameters of gait is interfered with body height [23]. Therefore, both step length and width were normalized to body height (*bh*) [20, 24, 25]. The trunk angle was calculated between the trunk segment and a vertical axis in the sagittal plane. The orientation of the trunk segment was represented by a line connecting the middle point of the hips and the middle point of the shoulders. Positive trunk angle denotes that the trunk leans backward against the vertical line. The trunk angle was computed at both TD and LO. Gait speed was also calculated as the average value of the calculated instantaneous COM velocity over an entire gait cycle and was normalized to *bh*.

The body COM kinematics were computed using gender-dependent segmental inertial parameters [26]. The two components of the COM motion state, i.e. its position and velocity were calculated relative to the rear of BOS (i.e. the leading heel) and normalized by foot length (*l*_BOS_) and $\sqrt{g\times bh}$, respectively, where *g* is the gravitational acceleration. Dynamic gait stability was calculated as the shortest distance from the given COM motion state to the limit against backward balance (Fig 1) [27]. The COM motion state and dynamic gait stability were calculated at instants of TD and LO.

### Statistical Analysis

All statistics were performed using SPSS 22.0 (IBM, NY), and a significance level of 0.05 was used throughout. Independent *t*-tests were used to compare the temporal and spatial parameters between the two groups: normal-weight vs. obese. Temporal parameters included the durations of single- and double-stance phases, step time, and cadence. The spatial parameters consisted of the step length, step width, gait speed, trunk angle, the COM motion state and dynamic stability at both TD and LO. The effect size (Cohen’s *d*) was also calculated for each parameter to indicate the magnitude of the difference between groups.

## Results

**Fig 2:**
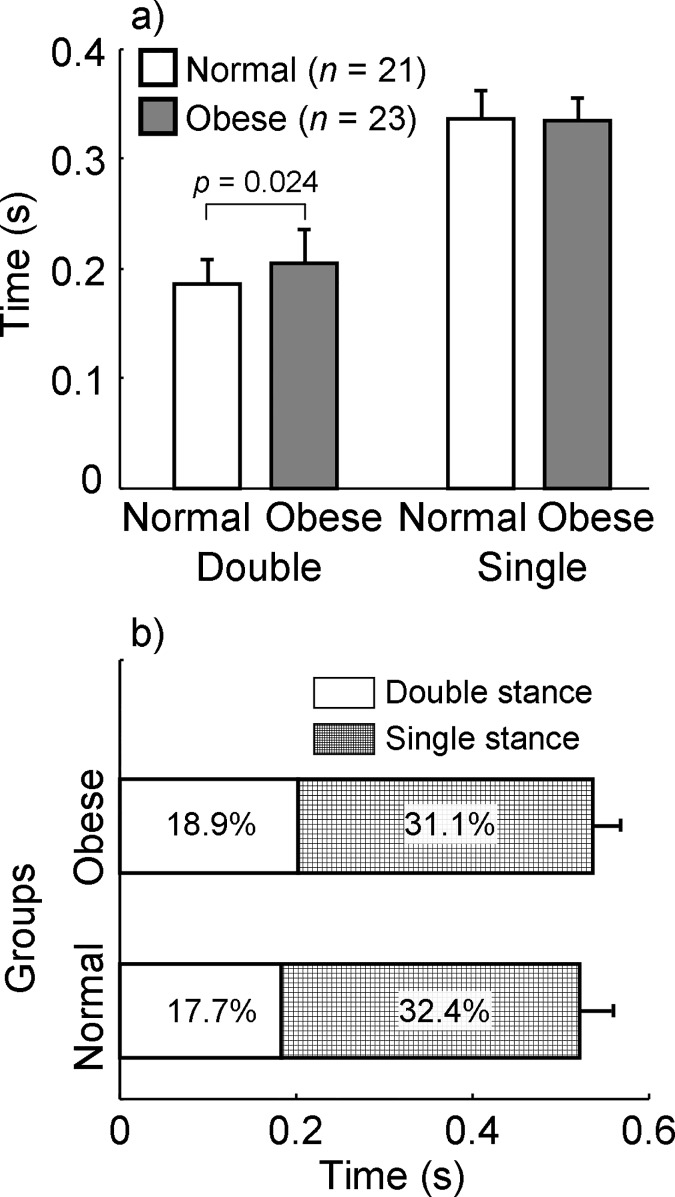
Group mean (column height) and standard deviation (error bar) of the elapsed time in seconds for both normal-weight (or Normal) and obese (or Obese) groups of (a) double and single stance phases and (b) the step time, defined as the duration from touchdown of one foot to the following touchdown of the contralateral foot (i.e., the sum of the single and double phases). Also shown are the percentages of the single stance and double stance with respect to the gait cycle.

**Fig 3:**
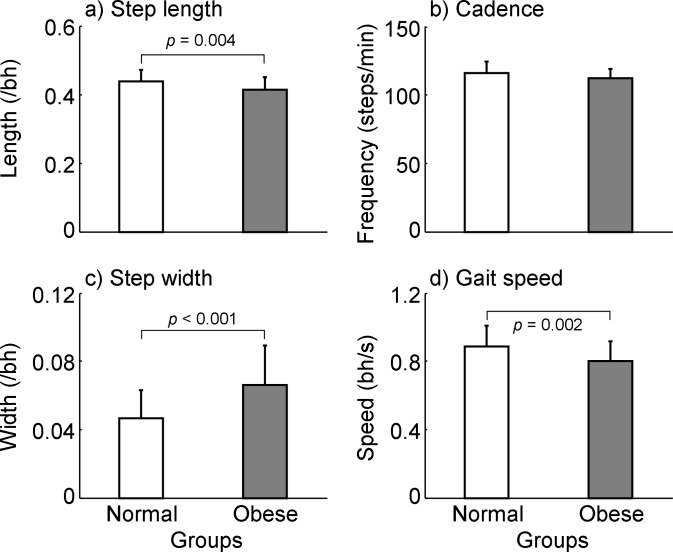
Comparisons of (a) the step length, (b) cadence, (c) step width, and (d) gait speed between normal-weight and obese groups. The step length/width was calculated as the anteroposterior/mediolateral distance between two heels at their touchdowns and normalized to the body height (*bh*). The cadence was determined as the reciprocal of the step time and expressed over one minute. The gait speed was the average value of the instantaneous center of mass velocity over the entire gait cycle and normalized to *bh*. The center of mass velocity was calculated as the first-order derivative of the center of mass displacement with respect to time.

When allowed to walk at their self-selected pace, people with obesity spent longer time during the double stance phase than those with normal weight (0.204 ± 0.032 *s* for obese vs. 0.185 ± 0.023 *s* for normal-weight, *p* = 0.030, Cohen’s *d* = 0.647, Fig 2A) while their duration of the single stance phase was comparable (0.337 ± 0.025 vs. 0.334 ± 0.020 *s*, *p* = 0.715, *d* = 0.112, Fig 2A). The relative double stance phase to the gait cycle was also longer among obese than in normal-weight (18.9 ± 2.31% vs. 17.7 ± 1.42%, *p* = 0.050, Fig 2B). The step time did not differ between groups (0.521 ± 0.037 vs. 0.538 ± 0.029 *s*, *p* = 0.111, *d* = 0.483, Fig 2B), neither did the cadence (112.40 ± 7.12 vs. 116.10 ± 8.34 steps/min, *p* = 0.121, *d* = 0.470, Fig 3B). Persons with obesity walked more slowly than normal-weight individuals (0.792 ± 0.098 vs. 0.895 ± 0.111 *bh*/*s*, *p* = 0.002, *d* = 0.885, Fig 3A; or 1.352 ± 0.174 vs. 1.463 ± 0.182 m/s, *p* = 0.045, *d* = 0.602) with a shorter step length (0.411 ± 0.029 vs. 0.440 ± 0.034 *bh*, *p* = 0.004, *d* = 0.839, Fig 3C) and a wider step width (0.070 ± 0.025 vs. 0.046 ± 0.017 *bh*, *p* < 0.001, *d* = 0.974, Fig 3D).

**Fig 4:**
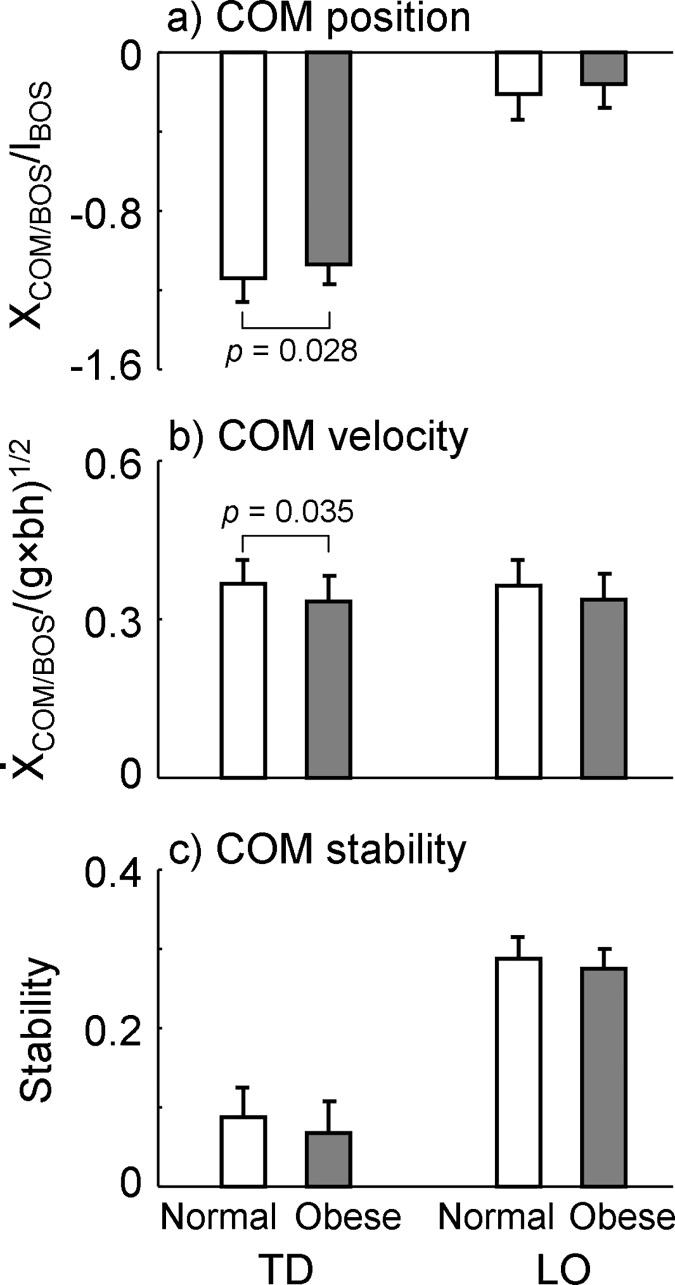
Comparisons of (a) the center of mass (COM) position, (b) COM velocity, and (c) COM stability at two transient gait events (touchdown or TD and liftoff or LO) between normal-weight and obese groups. Both the COM position and velocity were relative to the rear edge of the base of support (BOS) and respectively normalized by foot length (*l*_BOS_) and $\sqrt{g\times bh}$, where *g* represents the gravitational acceleration and *bh* the body height. Stability is calculated as the shortest distance from the given COM motion state (i.e. its position and velocity) and the computer-predicted boundary against backward balance loss (Fig 1).

**Fig 5:**
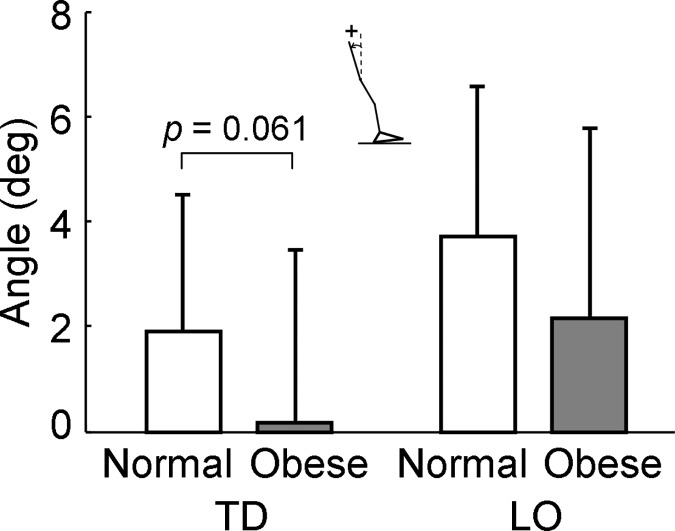
The comparison of the trunk angle at two transient gait events (touchdown or TD and liftoff or LO) between normal-weight and obese groups. Trunk angle was calculated between the trunk segment and a vertical axis. Positive trunk angle represents that the trunk leans backward against the vertical line while zero means the trunk is in a neutral position which aligns perfectly with the vertical axis.

Participants placed their COM more forward towards the BOS in the obese group than in the normal-weight group at the beginning (i.e., at TD, -1.071 ± 0.105 vs. -1.146 ± 0.114, *p* = 0.028, *d* = 0.655, Fig 4A) but not at the end of the stance phase (at LO, -0.165 ± 0.122 vs. -0.212 ± 0.137, *p* = 0.233, *d* = 0.363, Fig 4A). The relative COM velocity to the BOS was slower among people with obesity compared to those with normal weight at TD (0.333 ± 0.047 vs. 0.364 ± 0.046, *p* = 0.035, *d* = 0.630, Fig 4B) while the COM velocity did not exhibit any group-related difference at LO (0.336 ± 0.049 vs. 0.361 ± 0.050, *p* = 0.095, *d* = 0.505, Fig 4B). The dynamic gait stability against backward balance loss did not show any difference between groups (TD: 0.067 ± 0.040 vs. 0.086 ± 0.038, *p* = 0.108, *d* = 0.486; LO: 0.274 ± 0.026 vs. 0.286 ± 0.027, *p* = 0.147, *d* = 0.440, Fig 4C). At TD, the obese group leaned their trunk backward for 0.181 ± 3.275° from the vertical line which was marginally less than the one (1.905 ± 2.596°) among the normal-weight group (*p* = 0.061, *d* = 0.563). The trunk angle was alike between groups at LO (2.153 ± 3.623 vs. 3.698 ± 2.875°, *p* = 0.127, *d* = 0.462, Fig 5). All original data was provided in the supporting file (S1 File).

## Discussion

This study sought to determine how obesity affects dynamic stability during gait among young adults. Our results indicated that people with obesity walked more slowly, with a shorter but wider step, and elongated double stance phase in comparison with normal-weight people when they had the option of walking at a self-chosen gait speed. As a result of these adaptive changes to the gait pattern, people with obesity displayed similar dynamic gait stability to those with normal weight, which supported our hypothesis.

Consistent with previous findings [18–20], people with obesity in the present study demonstrated a slower self-selected gait speed, a shorter step length, a wider step width, accompanied by a prolonged double stance phase (Figs 2 and 3). Except the step width, other adaptive changes to the gait pattern exhibited in the obese group have been linked to the attempt of reducing energetic costs [28]. It was suggested that the preferred gait variables were chosen by obese young adults to minimize their mechanical work required to transfer the excessive body mass [28]. This was supported by another study indicating that both obese and normal-weight individuals preferred to walk at speeds where the gross energy cost per distance was minimized [19]. To move the excessive body mass, people with obesity may choose a slower gait speed as a strategy to expend possibly minimal energy. Particularly, the slow walking speed reduces the mechanical work demanded to redirect the COM of forward or upward movements during gait. In the current study, individuals with obesity do not reduce the step frequency but step length to slow the gait speed (Fig 3), which confirms previous findings [29]. A possible explanation from the energy efficiency perspective could be that a shorter step may dissipate less energy and reduce the energy expenditure [30].

These modifications to the gait spatiotemporal parameters, which have previously been reported as safety-related adaptations [31], are indications of the “cautious gait” used by obese participants. These adaptive gait pattern changes were also suggested being indicative of reaction to maintain dynamic stability during gait [19]. A slow gait speed possibly allows people with obesity a more controlled gait pattern in order to maintain dynamic balance. There could be other factors contributing to the slow gait speed among people with obesity. First, obese individuals generally experience lower muscle strengths than their normal-weight counterparts when adjusting for body mass [32]. The relative muscle weakness in people with obesity could result in slow gait speed [33]. Second, the degraded plantar sole sensitivity level among the obese may delay the detection of external perturbations and elevate the risk of experiencing an actual fall should a perturbation occur. A secure and more controlled gait pattern would allow more time for sensory exploration, attenuate the sensation of uncertainty, and potentially reduce the severity of any possible perturbations. Thus, the cautious gait pattern also compensates, to some extent, the sole sensory impairments in obese [3].

In terms of the FSR theory, a reduced gait speed, leading to a decreased COM velocity relative to the BOS, would compromise dynamic gait stability in people with obesity [27]. To compensate such a negative influence on stability from the reduced gait speed, people with obesity adopted a short step during walking. They shortened their step length by approximately 6% in comparison with their normal-weight counterparts (Fig 3A). It has been found that a reduced step length, which shifts body COM anteriorly and close to the BOS, improves stability against backward falls [21]. When the COM is close to the BOS, less forward momentum is required to enable the COM to catch BOS in order to keep body balance [34]. Therefore, the anteriorly-shifted COM will counter the reduction in stability resulting from the slow COM velocity.

The modification in participants’ COM position was also achieved by reducing the backward trunk lean (Fig 5). Less trunk backward inclination further shifts the COM forward [21, 34] given that the HAT (head, arm, and trunk) segment contains about two thirds of the total body mass. Such an effect would be magnified by the excess HAT segment mass in people with obesity. The anterior shift of COM could reduce the required COM velocity and warrant the dynamic stability against backward balance loss [34].

A healthy gait pattern is governed by the central nervous system for both economy and stability. A previous study has proposed that the metabolic rate and mechanical efficiency are comparable between obese and normal-weight groups when allowed to walk at self-selected gait speed [28]. Another study seconded this finding by showing that no significant difference in the external work-per-unit mass was detected between obese and normal-weight groups when walking at a comfortable gait speed [35]. The present study further demonstrated that people with obesity demonstrate similar dynamic stability during gait to those with normal weight (Fig 4C). People with obesity adaptively changed their gait pattern in response to the excessive gait mass in order to minimize the metabolic cost and to sustain dynamic stability. Therefore, the central nervous system regulates the obese gait to be economically and mechanically comparable with the non-obese gait.

Despite studies which pointed out that obesity could reduce the static stability quantified by the postural sway when standing on force plates, obesity seems not affecting the dynamic stability during gait when subjects are allowed to walk at their preferred speeds. Given that the bipedal human motion is inherently instable due to its multi-link inverted pendulum structure [36], the control of stability is essentially a matter of regulating the relative motion between the COM and its BOS [37]. During static tasks like quiet standing, the relative position between the COM and BOS plays the dominant role in controlling balance [37]. To keep the body balanced, one needs to keep the projection of the COM within the BOS. As aforementioned, obese individuals possess excessive body mass with relatively weak muscle strength [33]. Thus, people with obesity may encounter difficulties in confining the body’s sway within a range which is as small as among normal-weight individuals. As a result, obese individuals exhibit a greater body sway–the indicator of postural instability. In contrast, the regulation of the relationship between COM and BOS in gait is highly complex because the BOS and the COM are in constant motion with the BOS changing its size. It has been reported that a person controls simultaneously the COM position and velocity relative to the BOS in order to avoid a balance loss during gait [14, 15]. Therefore, two dimensions (i.e., the position and velocity) of two elements (i.e., the COM and BOS), or more degrees of freedom than static situation, could be manipulated to maintain dynamic stability. In the present study, people with obesity adopted a slower speed to reduce the COM velocity relative to the BOS and a shorter step length and less backward-leaning trunk to bring the COM closer to the BOS than those with normal weight. The dynamic stability did not demonstrate any significant difference between groups. Those adaptive changes to the gait pattern could be considered actions to maintain gait stability in obese individuals [19].

In line with previous ones [18–20], the current study found adaptive changes to the gait pattern among people with obesity, such as the reduced gait speed, shortened step length, widened step width, and prolonged double stance phase. The dependency relationship of spatial gait parameters upon gait speed was reported previously. For example, when one slows the gait velocity, a shorter step would be taken [38]. It was suggested that people with obesity tend to walk slowly to preserve dynamic balance [19]. Therefore, a question may rise: are other changes detected in the current study also solely owning to obesity or the secondary (or compensatory) outcomes of the slow speed? To answer this question, more systematical and comprehensive studies using biomechanical and physiological approaches are essential.

Several limitations were presented in this study. First, the walking condition involved in this study was normal overground walking. As most falls are initiated by external perturbations (such as slips or trips), it is unknown if the finding from this study can be applied to a perturbed walking condition. Second, the sample size was small. This could partially explain the non-significant difference in few parameters including the cadence (Fig 3B) and trunk angle at TD (Fig 5) although they exhibited a close to moderate effect size. Third, the COM kinematics calculation was based on the same segmental inertial parameters for both groups [26]. This system may not account for the potential anterior shift of the COM due to the additional abdominal fat in obese [39]. However, the current calculation of the COM position for the individuals with obesity was a conservative estimation. If appropriate adjustments were made to the COM kinematics calculation among obese, the COM would be more anteriorly shifted than the current values, which would still be in favor of our findings. Fourth, obesity could impact human body stability on both anteroposterior and mediolateral directions [6–8]. Although the present study examined the impact of obesity on dynamic stability control in the sagittal plane, it is unknown how dynamic stability behaves on the frontal plane in people with obesity. Fifth, the physical activity level was not controlled well between groups in this study. It is possible that the physical activity level could be a confounder influencing our findings. Last, some parameters, such as the waist to hip ratio and abdominal circumference were not measured in the present study, which limit our ability of determining the fat distribution type and its potential influences on our findings. All issues deserve further investigations.

In summary, this study advanced the examination of stability control from a static condition to a dynamic one among people with obesity. Our results revealed that people with obesity have similar dynamic stability during gait as those with normal weight. The comparable dynamic stability is the compounding effects of the reduced gait speed and shortened step length along with a less backward-leaning trunk segment among the obese group than the normal-weight group. The findings of this study could be of importance to examine the control of dynamic stability among people with obesity.

## Supporting Information

S1 File All original data used in the manuscript. This file contains all data utilized in the manuscript.
(XLSX)
